# Multiplexed Single Intact Cell Droplet Digital PCR (MuSIC ddPCR) Method for Specific Detection of Enterohemorrhagic *E. coli* (EHEC) in Food Enrichment Cultures

**DOI:** 10.3389/fmicb.2017.00332

**Published:** 2017-03-02

**Authors:** Tanis C. McMahon, Burton W. Blais, Alex Wong, Catherine D. Carrillo

**Affiliations:** ^1^Research and Development, Ottawa Laboratory (Carling), Ontario Laboratory Network, Canadian Food Inspection Agency, OttawaON, Canada; ^2^Department of Biology, Carleton University, OttawaON, Canada

**Keywords:** STEC, intimin, EHEC, Shiga toxin, droplet digital PCR, quantitative PCR

## Abstract

Foodborne illness attributed to enterohemorrhagic *E. coli* (EHEC), a highly pathogenic subset of Shiga toxin-producing *E. coli* (STEC), is increasingly recognized as a significant public health issue. Current microbiological methods for identification of EHEC in foods often use PCR-based approaches to screen enrichment broth cultures for characteristic gene markers [i.e., Shiga toxin (*stx*) and intimin (*eae*)]. However, false positives arise when complex food matrices, such as beef, contain mixtures of *eae*-negative STEC and *eae*-positive *E. coli*, but no EHEC with both markers in a single cell. To reduce false-positive detection of EHEC in food enrichment samples, a Multiplexed, Single Intact Cell droplet digital PCR (MuSIC ddPCR) assay capable of detecting the co-occurrence of the *stx* and *eae* genes in a single bacterial cell was developed. This method requires: (1) dispersal of intact bacteria into droplets; (2) release of genomic DNA (gDNA) by heat lysis; and (3) amplification and detection of genetic targets (*stx* and *eae*) using standard TaqMan chemistries with ddPCR. Performance of the method was tested with panels of EHEC and non-target *E. coli*. By determining the linkage (i.e., the proportion of droplets in which *stx* and *eae* targets were both amplified), samples containing EHEC (typically greater than 20% linkage) could be distinguished from samples containing mixtures of *eae*-negative STEC and *eae*-positive *E. coli* (0–2% linkage). The use of intact cells was necessary as this linkage was not observed with gDNA extracts. EHEC could be accurately identified in enrichment broth cultures containing excess amounts of background *E. coli* and in enrichment cultures derived from ground beef/pork and leafy-green produce samples. To our knowledge, this is the first report of dual-target detection in single bacterial cells using ddPCR. The application of MuSIC ddPCR to enrichment-culture screening would reduce false-positives, thereby improving the cost, speed, and accuracy of current methods for EHEC detection in foods.

## Introduction

Foodborne illness due to Shiga toxin-producing *E. coli* (STEC) continues to be an important public health concern in Canada and around the world (EFSA, 2013; Gould et al., 2013; Thomas et al., 2015). A sub-group of STEC, the enterohemorrhagic *E. coli* (EHEC), causes infections that can result in serious medical conditions including bloody diarrhea, hemolytic-uremic syndrome (HUS), kidney failure and microangiopathic hemolytic anemia, and can occasionally be fatal (Karmali et al., 2010; Thomas et al., 2015). Consumption of foods contaminated with EHEC is an important cause of illnesses associated with this pathogen. High-risk foods, such as ground beef and produce, are thought to become contaminated through exposure to animal fecal matter, particularly from ruminant animals in which STEC bacteria are prevalent (Gill and Gill, 2010; Mathusa et al., 2010; Duffy et al., 2014). Surveillance and recall of EHEC-contaminated foods reduces risk for the consumer, and improved methods will enable more extensive testing and further reduce human-health risk attributed to this organism (EFSA, 2013; Catford et al., 2014; Duffy et al., 2014; Seys et al., 2015).

The most common EHEC is *E. coli* O157, which has been the focus of public health organizations. However, non-O157 EHEC foodborne illnesses have been increasingly identified (Johnson et al., 2006; Gould et al., 2013; Catford et al., 2014; Luna-Gierke et al., 2014). There are no biochemical features by which EHEC strains can be differentiated from commensal *E. coli* or other STEC that are not a public health concern. Nonetheless, it is universally recognized that foodborne EHEC can generally be defined on the basis of certain gene markers, including the Shiga toxin genes, *stx1* or *stx2*, the intimin-coding gene, *eae*, along with markers for specific serogroups of concern (e.g., O26, O45, O103, O104, O111, O121, O145, and O157) (Blais et al., 2012; EFSA, 2013; Catford et al., 2014). Note that while most EHEC strains have both *eae* and *stx* genes, priority serogroups vary among countries.

The method for detection and isolation of EHEC used in food-testing laboratories at the Canadian Food Inspection Agency (Gill et al., 2012; Huszczynski et al., 2013; Blais et al., 2014a,b) as well as the ISO/CEN TS13136:2012 Technical specification (ISO, 2012) and the US MLG5B.05 (USDA-FSIS, 2014) methods, commonly used internationally, involve enrichment of samples in a selective broth and screening for the presumptive presence of EHEC using PCR methods targeting *stx1* and/or *stx2*, and *eae*. One of the challenges of screening enrichment broths for EHEC is to distinguish samples with target EHEC carrying both *stx* and *eae* from samples containing mixed cultures in which these markers are present in different cells. Using current approaches, as much as 50% of samples identified as presumptive positives may be false positives, particularly in samples with high levels of non-target *E. coli* (Livezey et al., 2015; Delannoy et al., 2016). This high rate of false positives can negate the benefit of a screening procedure intended to identify presumptive EHEC (i.e., *E. coli* cells carrying both *stx* and *eae*) due to the need for unnecessary downstream processing of samples for the recovery and characterization of the target bacteria.

**FIGURE 1:**
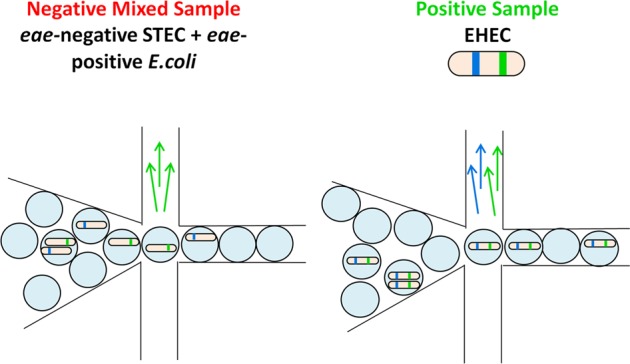
**Droplet Digital PCR Method for specific detection of intact EHEC cells.** Samples of food enrichment broths containing intact cells are added to the PCR reaction. A 20 μL PCR reaction is converted into 20,000 1 nL droplets prior to PCR-amplification. Amplification of *eae* (green) and *stx* (blue) is determined based on detection of fluorescent probes for each assay. In samples containing EHEC, both *eae* and *stx* targets will be detected in positive droplets. In samples containing mixtures of *eae*-negative STEC and *eae*-positive *E. coli*, most droplets will contain either *eae* or *stx*, unless both bacteria types are present within a single droplet.

The aim of this work was to develop a screening method capable of distinguishing enrichments positive for EHEC from false-positive samples containing mixed cultures of *eae*-negative STEC and *eae*-positive *E. coli* using droplet digital PCR (ddPCR) technology (**Figure 1**). ddPCR is a quantitative PCR technique in which a standard PCR reaction mixture is distributed into thousands to millions of droplets prior to PCR amplification (Hindson et al., 2011; Pinheiro et al., 2012). The Bio-Rad QX200^TM^ implementation of this technology involves conversion of 20 μL reaction volumes into approximately 20 thousand one-nanoliter droplets followed by PCR amplification within each droplet. The concentration of the input sample is adjusted to achieve a distribution of less than one template molecule per droplet with a Poisson distribution of the template molecules among droplets (Pinheiro et al., 2012). TaqMan probe-based PCR assays are commonly used in this method, and amplification is determined by detection of fluorescence in each droplet by a droplet reader. The number of positive droplets indicates the number of template molecules in the sample and linkage between different targets (e.g., *eae, stx*) can be determined based on frequency of co-amplification of two targets within a droplet. In samples containing EHEC, co-amplification of *eae*/*stx* targets would be expected to be high; whereas in false-positive samples (i.e., mixtures of *eae*-negative STEC and *eae*-positive *E. coli*) the two targets would generally be amplified in separate droplets, with only a small number of droplets in which both targets were amplified due to presence of two different bacterial cells.

Here we describe the development of a Multiplexed, Single Intact Cell ddPCR (MuSIC ddPCR) assay targeting detection of *stx* and *eae* genes and ensuring presence of the two targets within a single bacterial cell. In this assay, suspensions of bacterial cells in enrichment broth are dispersed into droplets, followed by release of gDNA by heat lysis, and ddPCR analysis. The method was evaluated to determine performance for specific identification of EHEC in enrichment-broth samples containing excess amounts of non-target bacteria.

## Materials and Methods

### Growth and Maintenance of *E. coli* Strains

A selection of *E. coli* strains of various serotype, *eae*, and *stx* gene profiles were used to evaluate the MuSIC ddPCR method (**Table 1**). An *E. coli* strain lacking the *stx* and *eae* genes was used as a negative control (OLC1543) and a strain containing a plasmid encoding fragments of the *stx1, stx2* and *eae* genes was used as a positive control (OLC2283, see description below). All strains were stored at -80°C in 25% glycerol and were plated on Brain-Heart Infusion agar (BHI) (OXOID, Nepean, ON, Canada) overnight (14–16 h) at 37°C prior to use. Samples were prepared for ddPCR experiments by transferring growth from a single colony into 10 mL of nutrient broth (OXOID) or modified Tryptone Soya Broth (mTSB)(OXOID) and growing overnight at 37°C. Prior to use, broths were diluted in nutrient broth (OXOID) to approximately 100 cells/μL. The strains used to generate mixed-culture samples were grown separately and mixed in equal amounts before diluting. Bacterial concentrations in overnight cultures (nutrient or mTSB broth) were initially determined by duplicate plating of serial dilutions on BHI agar. Subsequently, the A_600_ absorbance was used to estimate concentrations. In general, STEC strains reached a concentration of approximately 10^8^–10^9^ cells/mL after an overnight growth at 37°C. The plasmid control (OLC2283) and the generic *E. coli* (OLC1543) reached concentrations of approximately 10^9^–10^10^ cells/mL.

**Table 1:** List of *E. coli* strains used in this study.

Strain name	Serotype (toxin profile)	*stx*	*eae*
OLC0455^1^	O111:H11 (*stx1a*)	+	+
OLC0456	O111:H8 (*stx1a*)	+	+
OLC0464^1^	O26:H11 (*stx1a*)	+	+
OLC0467^3^	O5:NM (*stx1a*)	+	+
OLC0639^2^	O26:H11 (*stx1a*)	+	+
OLC0669^2^	O76:H19 (*stx1*)	+	-
OLC0675^1^	O145:NM (*stx1a*)	+	+
OLC0679^1^	O103:H2 (*stx1a*)	+	+
OLC0684^1^	O145:NM	-	+
OLC0710^1^	O121:H19 (*stx2a*)	+	+
OLC0716^1^	O45:H2 (*stx1a*)	+	+
OLC0728^1^	O103:H11 (*stx1a*)	+	+
OLC0797^1^	O157:H7 (*stx1a, stx2a*)	+	+
OLC0986	O157:H7 (*stx2a, stx2c*)	+	+
OLC0997^2^	O118:H12 (*stx2b*)	+	-
OLC0998^2^	O73:H18 (*stx2d*)	+	-
OLC0999^2^	O2:H25 (*stx2g*)	+	-
OLC1001^2^	O128ac:H2 (*stx2f*)	+	+
OLC1002^2^	O174:H8 (*stx1c, stx2b*)	+	-
OLC1003^2^	O139:K12:H1 (*stx2e*)	+	-
OLC1059	O157:H7 (*stx2c*)	+	+
OLC1060	O166:H15 (*stx2d*)	+	-
OLC1069	O121:H19 (*stx2a*)	+	+
OLC1070	O157:H7 (*stx2a, stx2c*)	+	+
OLC1251	O91:H14 (*stx2b*)	+	-
OLC1254	O166:H15 (*stx2d*)	+	-
OLC1256	O55:H7 (*stx1a*)	+	+
OLC1258	O145:H34 (*stx2f*)	+	+
OLC1263	O26:H11 (*stx2a*)	+	+
OLC1267	O8:H10 (*stx2e*)	+	-
OLC1269	O2:H25 (*stx2g*)	+	-
OLC1335	O154:H31(*stx1d*)	+	-
OLC1535	O185:H7 (*stx2c*)	+	-
OLC1685	OUT:H23 (*stx2e*)	+	-
OLC2238	O159:H19 (*stx2a*)	+	-
OLC2250	O91:H14 (*stx1a*)	+	-
OLC2284	O157:H7 (*stx2c*)	+	+
OLC2285	O157:H7 (*stx1a, stx2a*)	+	+
OLC1543 (negative control)	O87:H7	-	-
OLC2283 (positive control)	-	Fragment	Fragment

*^1^Strains were previously described in Lambert et al. (2015). ^2^Strains were previously described in Blais et al. (2014a). ^3^Strains were previously described in Knowles et al. (2015). ^4^OUT indicates O-untypeable.*

### Preparation of Positive-Control Plasmid and Strain

A plasmid control was constructed by Integrated DNA Technologies (IDT, Coralville, IA, USA) using an artificial sequence designed to incorporate the sequences corresponding to the amplicons of the *stx1, stx2* and *eae* genes used in the ddPCR assay (Supplementary Figure 1). Sequences of the gene fragments integrated into the plasmid were based on the *E. coli* O157:H7 Sakai strain (Accession number: BA000007) (Hayashi et al., 2001). Fragments of other genes (*gyrB* and 16s rDNA) were inserted in between the *stx1, stx2* and *eae* genes for other applications. The sequence containing the gene fragments was inserted into the “Best-Fit” pIDTSMART-KAN Vector with a kanamycin marker (IDT), with an EcoRI restriction site at the 5′ end and BamHI restriction site target at the 3′ end of the artificial sequence.

The plasmid construct was transformed into *E. coli* DH5α cells using the Subcloning Efficiency^TM^ DH5α^TM^ Competent Cells (ThermoFisher Scientific Inc., Ottawa, ON, Canada), according to manufacturer’s instructions. Transformed cells were cultured on nutrient agar (OXOID) containing 50 μg/mL of kanamycin (Sigma, Markham, ON, Canada). Plasmid DNA was extracted using the Midi Plasmid Kit (Qiagen, Toronto, ON, Canada) and diluted to 5 fg/μL. Prior to use, the control strain was grown at 37°C overnight (14–16 h) on nutrient agar containing 50 μg/mL of kanamycin. Broth cultures were generated by transferring growth from a single colony into nutrient broth (OXOID) containing 50 μg/mL of kanamycin followed by overnight growth at 37°C.

### Primers and Probes

Primer and probe sequences for the *stx1, stx2* and *eae* genes were derived from the US Department of Agriculture’s non-O157 STEC Real-Time PCR (qPCR) Assay (Nielsen and Andersen, 2003; Perelle et al., 2004; Wasilenko et al., 2012; USDA-FSIS, 2014) (**Table 2**). Primers and Probes (IDT) were rehydrated to stock concentrations of 100 μM using 1X Tris-EDTA (TE) and stored at -20°C.

**Table 2:** Primers and probes used in this study.

Oligos	Sequence (5′ → 3′)	Amplicon size (bp)	Reference
**Primers**			
*stx*-F	TTT GTY ACT GTS ACA GCW GAA GCY TTA CG	129/133	Perelle et al., 2004
*stx*-R	CCC CAG TTC ARW GTR AGR TCM ACD TC		Perelle et al., 2004; Wasilenko et al., 2012
*eae*-F	CAT TGA TCA GGA TTT TTC TGG TGA TA	102	Nielsen and Andersen, 2003
*eae*-R	CTC ATG CGG AAA TAG CCG TTM		Nielsen and Andersen, 2003; Wasilenko et al., 2012
*stx1*-F	TTT GTT ACT GTG ACA GCT GAA GCT TTA CG	133	This paper
*stx1*-R	CCC CAG TTC AAT GTA AGA TCA ACA TC		This paper
*stx2*-F	TTT GTC ACT GTC ACA GCA GAA GCC TTA CG	129	This paper
*stx2*-R	CCC CAG TTC AGA GTG AGG TCC ACG TC		This paper
**Probes**			
*stx1*	56-FAM-CTG GAT GAT/zen/CTC AGT GGG CGT TCT TAT GTA A-3IABkFQ	–	Perelle et al., 2004; Wasilenko et al., 2012
*stx2*	56-FAM-TCG TCA GGC/zen/ACT GTC TGA AAC TGC TCC-3IABkFQ	–	Perelle et al., 2004; Wasilenko et al., 2012
*eae*	5MAXN-ATA GTC TCG CCA GTA TTC GCC ACC AAT ACC-3IABkFQ	–	Nielsen and Andersen, 2003; Wasilenko et al., 2012

*Mixed bases: Y (C,T), W (A,T), R (A,G), M (A,C), D (A,G,T), S (C,G).*

### Genomic DNA Extraction

Genomic DNA (gDNA) was extracted from 400 μL of culture grown in BHI broth for 3–4 h (OLC1543 was grown in nutrient broth) using the Maxwell^®^ 16 Cell DNA purification kit (Promega, Madison, WI, USA) according to manufacturer’s recommendations. The resulting gDNA was quantified using the Quant-iT^TM^ High-Sensitivity DNA assay kit (ThermoFisher Scientific Inc.) according to manufacturer’s recommendations and diluted to 5 pg/μL.

### Detection of EHEC in Samples Containing High Levels of Non-target *E. coli*

To simulate the impact of high backgrounds of non-target commensal bacteria, EHEC cells were added in different ratios (1:10, 1:100, 1:1000, 1:10,000) relative to the generic *E. coli* to a final concentration of approximately 10^9^ cells/mL. The STEC-*E. coli* samples were then diluted in nutrient broth as needed to reach a concentration of approximately 100 cells/μL of EHEC (10 cells/μL for the 1:10,000 dilution). For mixed cultures, overnight cultures of *eae*-negative STEC were mixed at a ratio of 1:1 with *eae*-positive *E. coli* prior to dilution as indicated above.

### Detection of EHEC in Food Enrichment Broths

To simulate EHEC enrichment in natural background flora found in raw meat and produce, 25 g of a mixture of ground beef and pork, or of samples of leafy-green produce (iceberg lettuce, kale, or spinach) were added to 225 mL of mTSB. Enrichment broths were incubated at 42°C for 16–18 h. Aliquots of 50 mL of the enrichment broths were centrifuged at 1500 × *g* for 1 min to remove debris followed by transfer of supernatants to new tubes. EHEC bacteria were then added to the background flora at proportions of 1:100 and 1:1000 (v/v). For mixed cultures overnight cultures of *eae*-negative STEC were mixed at a ratio of 1:1 (v/v) with *eae*-positive *E. coli* prior to diluting 1:10 (v/v) in enrichment broth.

### Quantitative PCR (qPCR)

Quantitative PCR was carried out using a Lightcycler 96 instrument (Roche Diagnostics, Laval, QC, Canada) according to manufacturer’s recommendations. Each 25 μL PCR reaction contained 12.5 μL of FastStart Essential DNA Probes Master (Roche Diagnostics), 1.25 μM of *stx*1 and *stx*2 primers, 1.0 μM of *eae* primers, 0.25 μM of *stx1* and *stx*2 probes, 0.2 μM of *eae* probe and 5 μL of 1 ng/μL of gDNA. The thermocycler conditions were as follows: one cycle of 95°C for 10 min; and 50 cycles of 94°C for 15 s and 55°C for 60 s. The data was analyzed using the Lightcycler 96 SW 1.1 Software.

### Droplet Digital PCR (ddPCR)

ddPCR was conducted using the QX200^TM^ ddPCR system (Bio-Rad, Mississauga, ON, Canada) according to the manufacturer’s recommendations. Each 25 μL PCR reaction mixture contained 1 X Supermix for Probes (Bio-Rad), 1.25 μM of *stx*, (or *stx*1 and *stx*2 primers), 1.0 μM of *eae* primers, 0.25 μM of *stx*1 and *stx*2 probes and 0.2 μM of *eae* probe. Approximately 500 intact cells or 25 pg gDNA was used in each assay (25 fg for plasmid DNA). An aliquot of 20 μL was taken from the 25 μL PCR reaction mix and loaded into a DG8 cartridge (Bio-Rad) with a volume of 70 μL of Droplet Generation Oil for Probes (Bio-Rad). The cartridge was placed in the Droplet Generator (Bio-Rad) to form the nanoliter droplets. For the droplet generation step, the Droplet Generator was placed in a Biosafety Cabinet as a precaution since the safety of generating droplets with intact pathogenic bacterial cells has not been studied. Droplets were then transferred to a 96 well plate that was sealed with foil using a PX1 PCR Plate sealer (Bio-Rad) prior to amplification using the C1000 Touch Thermocycler (Bio-Rad). The thermocycler conditions were as follows: one cycle of 95°C for 5 min; 40 cycles of 95°C for 20 s and between 51°C and 61°C for 60 s; one cycle of 94°C for 10 min; followed by cooling to 4°C. For reactions with restriction enzyme, 2.5 μL of BamHI FastDigest (ThermoFisher Scientific Inc.) were added to each PCR reaction and the following steps were added to the beginning of the thermocycler protocol: 80°C for 2 min (heat lysis); 37°C for 45 min (restriction digestion); and 80°C for 5 min (enzyme inactivation).

Following PCR amplification, samples were analyzed on the QX200 Droplet Reader (Bio-Rad) using the QuantaSoft^TM^ software (Bio-Rad). Samples with concentrations below 5 copies/μL and droplet counts below 10,000 droplets were discarded (Pinheiro et al., 2012). The linkage value generated by the QuantaSoft^TM^ software was used to assess association between the *eae* and *stx* targets; but, as this value is concentration-dependent, this linkage value was normalized by dividing by the concentration calculated for the *eae* assay and multiplying by 100 to generate a “percent linkage” value to enable comparison among experiments. The concentration of the *stx* assay was not used in the linkage normalization as there were differences in the number of *stx* genes among the isolates used in this study. Averages and standard deviations were determined for replicates.

## Results

### Development of the Multiplexed Single Intact Cell (MuSIC) ddPCR Method

#### Selection of Primers

**FIGURE 2:**
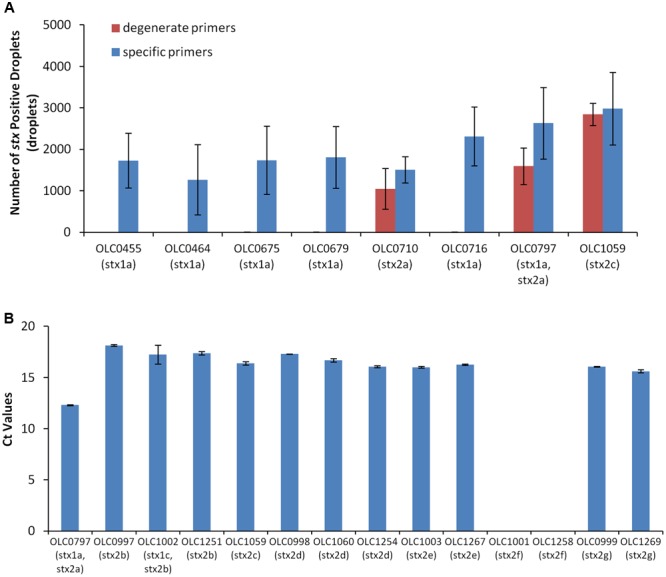
**Development of primers for the detection of Shiga Toxin genes. (A)** Use of degenerate primers (blue bars) in the ddPCR assay resulted in unreliable detection of *stx1* genes by ddPCR relative to the use of pools of specific primers (red bars) in strains with varying Shiga toxin profiles. Error bars represent standard deviations of 4 replicates. **(B)** Evaluation of the specific primers using qPCR demonstrated reliability of the assay with *stx2* subtypes *stx2a, stx2b, stx2c, stx2d, stx2e*, and *stx2g* but not *stx2f* (OLC1001 and OLC1258). Ct values were calculated using default parameters of the Lightcycler Software. Error bars represent the standard deviation of 2 replicates.

Primers and probes for *stx* and *eae* genes were based on those described in the MLG 5B method developed by the United States Department of Agriculture Food Safety and Inspection Service (USDA-FSIS, 2012) for detection of non-O157 STEC (MLG 5B, Appendix 1.01) (Nielsen and Andersen, 2003; Perelle et al., 2004; USDA-FSIS, 2012; Wasilenko et al., 2012). Due to the observation of lot-to-lot variations in the performance of these degenerate primers, described in an early implementation of the FSIS method, degenerate bases in the primers were removed and new primers specific to *stx1* or *stx2* were designed (**Table 2**). Performance of the specific primers relative to the degenerate primers was evaluated with a set of STEC with varying serological and toxin profiles using ddPCR (**Figure 2A**). Use of the specific primers resulted in higher numbers of positive droplets relative to results with degenerate primers, particularly for strains encoding *stx1* genes.

The specific primers were designed based on sequences for subtypes *stx1a* and *stx2a* of the *stx1* and *stx2* genes; however, there are three subtypes of *stx1* (*stx1a, stx1c, stx1d*) and seven subtypes of *stx2* (*stx2a, stx2b, stx2c, stx2d, stx2e, stx2f*, and *stx2g*). To evaluate the performance of the specific primers for detection of all of seven of the variants of the *stx2* genes, real-time PCR amplification of targets from STEC with various *stx2* subtypes was conducted (**Figure 2B**). The *stx2* genes were detected within all isolates with the exception of isolates encoding the *stx2f* subtype. By comparison of the primer sequences to gene sequences within a database of full-length Shiga toxin genes (vtx.fsa, updated 16 March 2016) from the Center for Genomic Epidemiology^1^ (Joensen et al., 2014), up to 3 mismatches to the specific *stx* primers were observed within the primer binding regions for most of the *stx2* genes. For the *stx2f* variants, up to 14 variable positions were observed for both the specific and degenerate primers. Conversely, the specific primers for *stx1* were generally 100% identical to sequences within *stx1a* subtypes, with only1–2 mismatches relative to sequences for *stx1c* and *stx1d* toxin subtypes.

An annealing temperature gradient experiment was performed to determine optimal temperatures for the new primers. Optimal distinction between the fluorescence amplitude intensity of positive and negative droplets was observed at temperatures between 54 and 57°C for the *stx* assay and between 56 and 59°C for the *eae* assay (data not shown). An annealing temperature of 56°C was selected for subsequent experiments.

### Comparison of the EHEC Multiplex ddPCR Using Intact EHEC Cells and Genomic DNA Extracts

**FIGURE 3:**
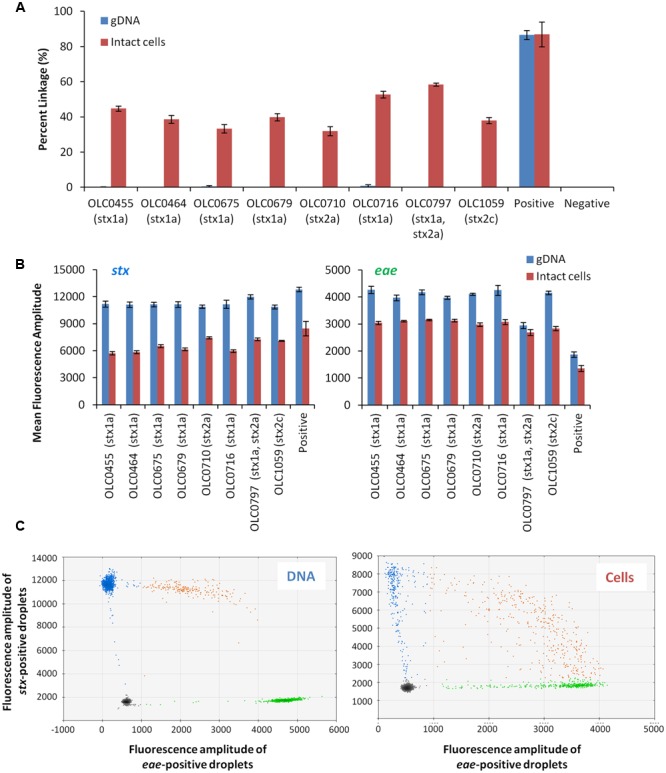
**Linkage between *stx* and *eae* genes in intact STEC cells, but not in extracted gDNA using ddPCR. (A)** Co-amplification of *stx* and *eae* in ddPCR was evaluated with a panel of EHEC strains in assays using gDNA as template (blue bars) and assays using whole cells as template (red bars). Association of EHEC virulence targets was assessed by calculating a normalized value for linkage, based on the values provided by the QuantaSoft^TM^ software (Percent linkage). A plasmid construct with both *stx* and *eae* genes or *E. coli* cells transformed with this plasmid (OLC2283), was used as a positive control. Error bars represent the standard deviation of 4 replicates. **(B)** For both *stx* (right panel) and *eae* (left panel) assays, mean fluorescence of positive droplets was significantly higher when gDNA was used as a template relative to results using intact cells. **(C)** Representative 2D plots generated by the QuantaSoft^TM^ analysis software for strain OLC0455 demonstrate more distinct partitions between positive and negative droplets when gDNA is used as a template (left panel) compared to intact cells (right panel). Droplets positive for *stx* amplification are blue, droplets positive for *eae* amplification are green, and droplets positive for both targets are orange.

To demonstrate the feasibility of using ddPCR with intact *E. coli* cells, a multiplex ddPCR experiment was conducted on a panel of STEC with various genetic profiles using both DNA extracts and intact cells (**Figure 3**). gDNA and overnight broth cultures were diluted to achieve appropriate concentrations for the ddPCR methods (∼500 cells or 25 pg/reaction). While amplification of the *eae* and *stx* targets was observed with both whole cell and gDNA extract templates, co-amplification of the two targets (based on linkage values determined from the QuantaSoft^TM^ program) was significantly higher (>31.8%) in reactions where intact cells were used as a PCR template compared to gDNA samples (<0.8%) (**Figure 3A**). In contrast, mean fluorescence amplitude of droplets in assays using gDNA template (**Figure 3B**) was higher, particularly for the *stx* target, than in assays where whole cells were used. Mean fluorescence amplitude was 6276 (cells) versus 11365 (gDNA) for the *stx* assay and 2820 (cells) vs. 3748 (gDNA) for the *eae* assay. This difference in fluorescence amplitude indicates that amplification efficiency may be impacted by the use of whole cells. Similarly, differences in fluorescence amplitude between positive and negative droplets were more distinct when gDNA was used as a template, relative to cells (**Figure 3C**). This was largely due to the increased mean fluorescence of the positive cells, as lower variability in the mean fluorescence intensity of the negative droplets was observed, with average mean fluorescence of 1685 (cells) and 1595 (gDNA) for the *stx* assay, and 520 (cells) and 532 (gDNA) for the *eae* assay.

Despite higher linkage of targets in whole-cell-based assays relative to assays using gDNA templates, the efficiency of dual-amplification of targets within droplets was lower than was predicted (**Figure 3C**, Cells). To determine if this was due to reagent limitation in the droplets and/or problems with the use of intact cells in the ddPCR assay, a positive control plasmid containing segments of *stx1, stx2* and *eae* was created (Supplementary Figure 1) and transformed into *E. coli* cells. Greater than 86% linkage of the *stx*/*eae* targets was observed when either the plasmid DNA or intact cells containing the control plasmid (OLC2283) was used as template in the ddPCR reaction (**Figure 3A**), indicating that the limitation in dual-target amplification within a single droplet was not due to the use of intact cells, or exhaustion of reagents.

#### Incorporation of Restriction Enzyme Digestion to Improve Droplet Separation

**FIGURE 4:**
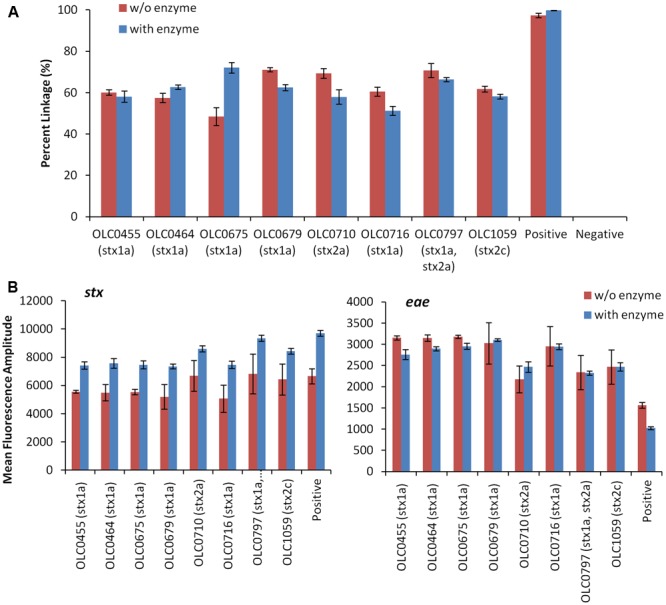
**Improved droplet separation with the addition of restriction enzyme in the MuSIC ddPCR assay. (A)** The integration of BamHI restriction enzyme digestion in MuSIC ddPCR was assessed using a panel of EHEC strains with various serological profiles. Association of EHEC virulence targets was assessed by calculating a normalized value for linkage, based on the values provided by the QuantaSoft^TM^ software (Percent linkage). Only minor differences in the proportion of droplets with amplification of both *stx*/*eae* targets was observed in assays with restriction enzyme (red bars) compared to reactions without restriction enzyme (blue bars). Error bars represent the standard deviation of 4 replicates. **(B)** An increase in the mean fluorescence amplitude of positive droplets for the *stx* assay (left panel) relative to assays conducted without the enzyme was observed. The use of the restriction enzyme did not seem to affect amplification of the *eae* target (right panel).

To assess the possibility that the tertiary structure of the chromosomal DNA was preventing access to the target genes, the impact of digestion of the DNA through the integration of the restriction enzyme BamHI in the ddPCR mixture was evaluated (**Figure 4**). While ideal reaction conditions for the restriction enzyme digestion could not be achieved, modifications to the PCR conditions were incorporated to favor DNA digestion (e.g., 45 min at 37°C). The addition of the restriction enzyme digestion did not result in an increase in the percent linkage between the *stx*/*eae* targets (**Figure 4A**). However, for the *stx* assay, there was an increase in mean fluorescence amplitude (8150 with enzyme versus 5818 without enzyme, **Figure 4B**) and more distinct partitions between positive and negative droplets. This difference in fluorescence amplitude was not observed for the *eae* gene (2548 with enzyme versus 2595 without enzyme). The mean fluorescence intensity of the negative droplets was slightly higher when enzyme was used, with a mean value of 1654 (enzyme) versus1563 (no enzyme) for the *stx* assay, and 603 (enzyme) versus 577 (no enzyme) for the *eae* assay, indicating a slight increase in autofluorescence in the enzyme assay. In both the *stx* and *eae* assays, standard deviation of the fluorescence amplitude among the four replicates was lower for the four samples in which enzyme was added. While the addition of enzyme does not seem to impact the linkage value used to identify EHEC samples, it was incorporated in the MuSIC ddPCR assay due to the observation of more efficient amplification of *stx* targets when the enzyme was incorporated.

### Evaluation of the MuSIC ddPCR Method

#### Distinguishing EHEC from Mixed Cultures of *eae*-Negative STEC and *eae*-Positive *E. coli*

**FIGURE 5:**
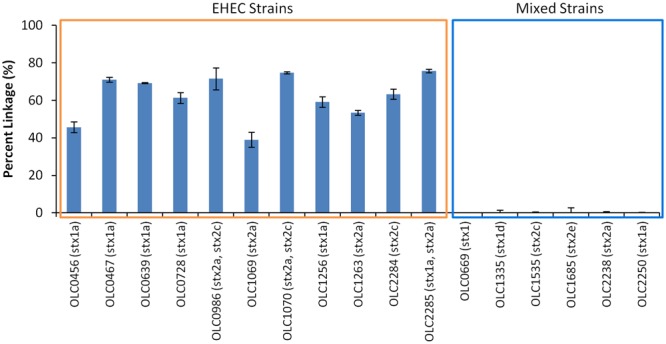
**Specific detection of EHEC and not mixtures of *eae*-negative STEC and *eae*-positive *E. coli* based on linkage of *eae* and *stx* targets using MuSIC ddPCR.** The performance of the MuSIC ddPCR assay was evaluated with a panel of 11 EHEC strains with varying serological profiles and with mixed cultures containing an *eae*-positive (OLC0684) with *eae*-negative STEC. Association of EHEC virulence targets was assessed by calculating a normalized value for linkage, based on the values provided by the QuantaSoft^TM^ software (Percent linkage). All positive samples had a percent linkage above 30% while the 6 mixed cultures did not show linkage between the targets. Error bars represent the standard deviation of 4 replicates.

Performance of the MuSIC ddPCR assay for distinguishing samples contaminated with EHEC from negative samples with mixtures of *eae*-negative STEC and *eae*-positive *E. coli* was evaluated. Overnight cultures of 11 EHEC strains (OLC0456, OLC0467, OLC0639, OLC0728, OLC0986, OLC1069, OLC1070, OLC1256, OLC1264, OLC2284, OLC2285) were diluted to concentrations of approximately 100 cfu/mL and 5 μL were added to each assay. For mixed cultures, overnight cultures of six strains of *eae*-negative STEC (OLC0669, OLC1335, OLC1535, OLC1685, OLC2238, OLC2250) were mixed at a 1:1 ratio with an overnight culture of an *eae*-positive *E. coli* (OLC0684), then diluted to approximately 100 cfu/mL. Samples containing EHEC could be accurately distinguished from mixed samples of *eae*-negative STEC and *eae*-positive *E. coli* based on the linkage between the *eae* and *stx* targets (**Figure 5**). This percent linkage was consistently above 39% for EHEC positive samples and below 1.99% for the mixed cultures. Concentrations of the EHEC and the mixed cultures used in these analyses were between 10 and 1300 cfu/μL (data not shown).

#### Performance of MuSIC ddPCR in Simulated Enrichment Broths

The MuSIC ddPCR assay is intended to be applied to the detection of EHEC in enrichment broths with high concentrations of bacteria (approximately 10^5^ cfu/μL), in which EHEC may be present at very low relative proportions (e.g., 0.01% of the population). Given that the maximum amount of enrichment broth that could be added to a ddPCR mixture is 5 μL, addition of undiluted enrichment broth to a ddPCR reaction (∼500,000 bacteria) would result in the compartmentalization of ∼25 non-target bacterial cells in each of the 20,000 droplets. To determine if high levels of non-target cells in each of the droplets would inhibit the MuSIC ddPCR assay, EHEC strains (OLC0464, OLC0710 and OLC0797) and mixed cultures (1:1) of *eae*-negative STEC (OLC1335 or OLC1535) and *eae*-positive *E. coli* (OLC0684) were combined with different amounts of a non-pathogenic *E. coli* strain (OLC1543). The final ratios of EHEC or mixed cultures relative to the background *E. coli* were 1:10, 1:100, 1:1,000, and 1:10,000. The STEC + background samples were diluted in nutrient broth to generate an optimal target concentration for the ddPCR assay (approximately 100 STEC/μL of sample or 1 STEC per 40 droplets) while maintaining the ratio of target cells to background. While the number of target cells remained consistent for each assay, the number of background cells per droplet varied, with up to 1 bacterial cell/4 droplets in the 1:10 sample, 2.5 cells/droplet in the 1:100 sample, and 25 cells/droplet in the 1:1000 and 1:10,000 samples. Lower concentrations of STEC (approximately 10 STEC/μL) were added for the 1:10,000 sample to avoid the need for a 10-fold concentration of the sample. For the mixed cultures, equal volumes of the overnight cultures were combined, resulting in lower concentrations of each of the targets for these samples as well (approximately 50 cfu of each strain/μL of the sample).

**FIGURE 6:**
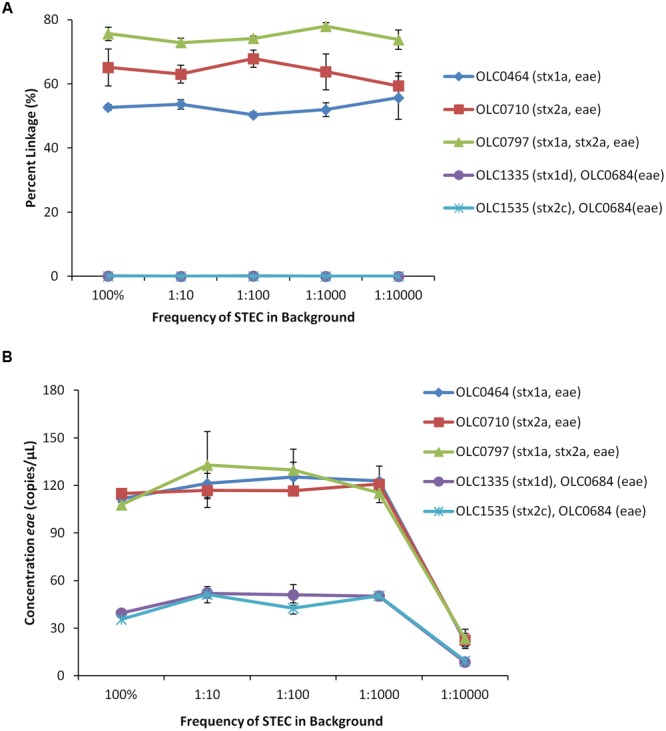
**Enterohemorrhagic *E. coli* strains accurately detected at relative proportions of 1 EHEC pathogen in 10,000 non-target *E. coli* (1:10,000) using MuSIC ddPCR. (A)** To simulate enrichment conditions, EHEC cultures were added to generic *E. coli* at relative proportions of 1:10, 1:100, 1:1000, and 1:10,000. Association of EHEC virulence targets was assessed by calculating a normalized value for linkage, based on the values provided by the QuantaSoft^TM^ software (Percent linkage). The EHEC samples were accurately detected (>50% linkage of *eae/stx*) in samples containing up to 10,000 non-target *E. coli* relative to target EHEC. The *eae/stx* genes were not linked in mixed cultures (OLC1335, OLC1535 mixed with OLC0684 (*eae*)) at any ratio. **(B)** Concentrations of the *eae* target in the samples determined based on values provided by the QuantaSoft^TM^ software. Error bars represent the standard deviation of 3 replicates.

For all of the EHEC samples, greater than 50% linkage between *eae* and *stx* targets was observed (**Figure 6A**). In contrast, in mixed samples of bacteria with targets in different cells, percent linkage values were below 0.15%. The concentration of the *eae* target in the EHEC strains was determined to be 115–130 copies/μL of test sample, with the exception of the 1:10,000 samples, which ranged in concentration between 21 and 40 copies/μL (**Figure 6B**). The concentration of the *eae* target in the mixed cultures was determined to be 38–52 copies/μL of test sample, with the exception of the 1:10,000 samples, which ranged in concentration between 8 and 9 copies/μL. These studies indicate that EHEC can be accurately detected in the presence of high levels of background microbiota, which could be presented in undiluted enrichment broths where target EHEC are present at relative concentrations as low as 1 target cell for every 10,000 non-target cells.

#### MuSIC ddPCR With Raw Ground Beef/Pork and Lettuce Enrichments

**FIGURE 7:**
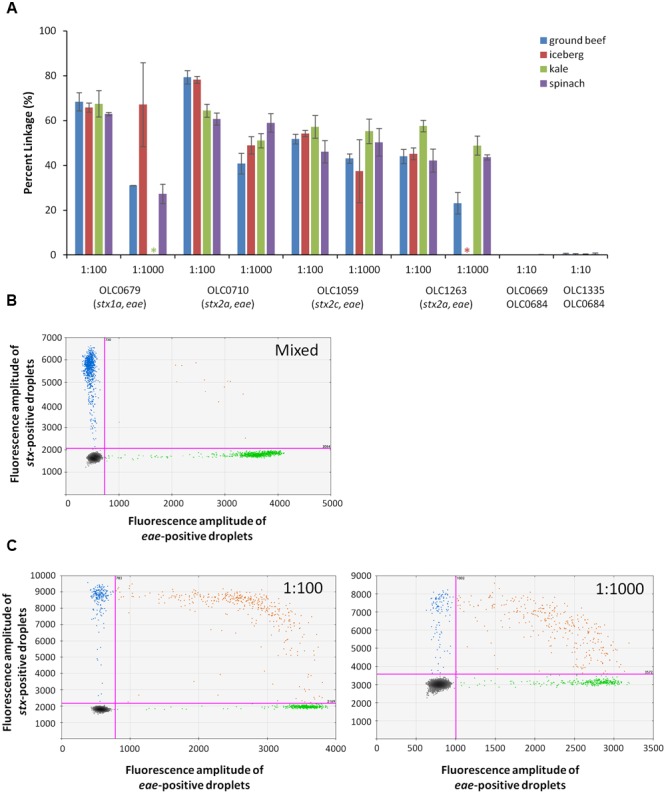
**Enterohemorrhagic *E. coli* accurately detected at concentration of 1 volume of STEC pathogen in 1000 volume of overnight beef and lettuce enrichment (1:1000) using MuSIC ddPCR. (A)** The STEC *eae* positive samples were positively detected with greater than 23% linkage for samples with as little as 0.1% of pathogen relative to non-target microorganisms in the ground beef/pork (blue), iceberg lettuce (red), kale (green) and spinach (purple) enrichment broths. Stars indicate samples that were excluded due to missing data. Error bars represent the standard deviation of 3 replicates. **(B)** A representative 2D plot generated by the QuantaSoft^TM^ analysis software for the 1:10 mixture of OLC0684 and OLC1335 with spinach enrichment broth is shown. Droplets positive for *stx* amplification are blue, droplets positive for *eae* amplification are green, and droplets positive for both targets are orange. **(C)** Representative 2D plots generated by the QuantaSoft analysis software for strain OLC1059 with spinach enrichment broth at relative proportions (target:background) of 1:100 (left) and 1:1000 (right) are shown.

Detection of EHEC in enrichment broths derived from food samples (ground beef/pork, leafy greens) was also evaluated to determine performance of the MuSIC ddPCR in the presence of typical food microbiota and inhibitors that may be present in these samples. Overnight cultures of EHEC strains OLC0679, OLC0710, OLC1059, and OLC1263 in mTSB were added to overnight ground beef/pork and produce enrichment broths at ratios of 1:100, 1:1000 (by volume). Mixed cultures of OLC0669 or OLC0335 with OLC0684 were diluted to relative proportions of 1:10 in the enrichment broths. Percent linkage between the *eae* and *stx* targets was greater than 23% in all of the 1:1000 EHEC samples, and greater than 43% in all of the 1:100 samples (**Figure 7A**). In contrast, in all of the mixed samples, percent linkage between these targets was lower than 0.45% (**Figures 7A,B**).

The concentration of the *eae* target was determined to be between 64 to 262 copies/μL of test sample in all samples included in the analysis (data not shown). Ratios of 1:1000 for samples OLC0679 in iceberg lettuce, and OLC1263 in kale were excluded from the analysis due to concentrations lower than 5 copies/μL. In spinach and kale samples, autofluorescence seemed to interfere with the *stx* assay when the enrichment broths were minimally diluted (**Figure 7C**). Mean fluorescence amplitude intensity of both negative and positive droplets was higher in the *stx* assay for EHEC samples with relative proportions of 1:1000 of EHEC to enrichment broth, than in samples with proportions of 1:100.

## Discussion

The MuSIC ddPCR assay represents a novel approach for the detection of two genetic targets within a single, intact, bacterial cell. To our knowledge, this is a new application of ddPCR technology. In this study, MuSIC ddPCR has been applied to the specific detection of EHEC in enrichment broths, and was shown to be able to distinguish samples contaminated with EHEC from samples containing mixed cultures of *eae*-negative STEC and *eae*-positive *E. coli*, even in samples with low proportions of target EHEC (e.g., 0.01% of the total bacterial population). This method is intended for samples in which target EHEC are present at high concentrations (e.g., 10–500 cfu/μL), and would be appropriate for screening enrichment broths derived from foods.

One of the early challenges in the development of this method was the lot-to-lot variability in the performance of the degenerate primers used in the original implementation of the method. While this degeneracy may not impact standard qPCR methods, for the ddPCR assay, the reduced concentrations of specific primers likely led to the exhaustion of these reagents in individual droplets. Specific primers performed more consistently, even in samples in which there were up to 3 mismatches between primers and primer binding sites. Note that this assay is not appropriate for the detection of the *stx2f* subtype of the *stx2* gene. Due to the high variability of the *stx2f* subtype relative to the other *stx2* subtypes, none of the methods currently deployed for the detection of EHEC are capable of detecting this subtype (Wasilenko et al., 2012; Blais et al., 2014b). Given the evidence that EHEC with this toxin variant are associated with clinical infections (Jenkins et al., 2003; van Duynhoven et al., 2008; Friesema et al., 2015), improvement of current methods through the addition of specific *stx2f* primers and probes may be of value.

The use of intact bacterial cells was required to detect linkage between the *eae*/*stx* targets indicative of the presence of EHEC. These targets were not linked when gDNA extracts were used as template in the assay, probably due to fragmentation of the chromosomal DNA during its preparation (**Figure 3**). The *stx* and *eae* genes are widely dispersed on the *E. coli* chromosome (typically over 1 Mb); therefore, it is highly probable that chromosomal breakage between these targets would occur during the extraction procedure (Brisco et al., 2010; Malentacchi et al., 2015), and that the fragments would segregate into different droplets. In the case of the intact cell assay, the contents of the entire cell would be present within a droplet, and linked targets would remain in the droplet, even if the gDNA became degraded after the cell lysis. This is consistent with the higher linkage values observed for intact cells compared to gDNA (**Figure 3**). Optimization of droplet ddPCR often requires development of conditions in which there is a clear distinction between positive and negative partitions (Huggett et al., 2013). While this partitioning could be easily achieved with the gDNA extracts, more variability in the fluorescence amplitude intensity was observed when cell cultures were used.

In the conception of this method, it was predicted that amplification of two targets would be observed for every droplet containing an intact EHEC cell. In practice though, dual amplification was lower than predicted, ranging from 23 to 79% of the droplets expected to be positive for both targets, with the lowest linkages observed for undiluted food enrichment broth samples. This was not due to reagent limitations, or the use of intact cells, as dual target amplification was observed in 87–99.9% of control samples of *E. coli* transformed with a plasmid containing both targets. Restriction digestion of gDNA templates is commonly recommended for ddPCR methods to relieve tertiary structure and improve target accessibility (Hindson et al., 2011) and so this approach was evaluated. While conditions for the restriction digestion were not optimal due to the need to apply heat to lyse cells at the beginning of the reaction, thereby partially inactivating the restriction enzymes, significant increases in the fluorescent intensities of the *stx*-positive droplets were observed (**Figure 4B**) indicating an improvement in the amplification efficiency. This improvement may be due to reduction in tertiary structure near the *stx* genes, improving accessibility target sites. In this study, this did not translate into an increased proportion of droplets in which dual-amplification of the targets was observed; nonetheless, the use of thermostable restriction enzymes should be explored to determine if further improvement to the partitioning of the positive and negative droplets could be achieved. Another possible explanation for the low proportion of droplets with dual-target amplification is that degraded DNA released from dead cells provided single-target templates for this reaction. Removal of free gDNA could potentially be achieved by adding a DNase step before the cell lysis. Although variability in percentage linkage was high in the experiments conducted in this study, linkage in non-target mixed culture samples was consistently less than 2%, and EHEC-positive samples could be easily identified using a conservative cut-off value of >10% linkage.

The use of cultures of intact bacterial cells and food enrichments in the MuSIC ddPCR assay instead of purified gDNA presented a number of challenges, likely due to presence of debris and particles in these samples. In this study, up to 6% of samples were lost due to blockages within capillaries which prevented droplet formation. In addition, droplet counts were not consistent. Under ideal conditions, droplet counts should approach 20,000, but when using cell cultures and enrichment broths, droplet counts varied from 10,000 to as high as 28,000. This is not surprising as the ddPCR reagents were developed for purified samples, and would not be expected to be optimal for these samples. Even with the use of purified gDNA, higher concentrations can increase viscosity of the sample, changing the average volume of the droplets (Hindson et al., 2011). In most cases, the problems with droplet generation were overcome with the use of 3–4 replicates per sample. The performance of the assay was also variable depending on the broth used for growth of the bacteria. Mean fluorescence amplitude was higher when cells were suspended in nutrient broth relative to BHI, mTSB and PBS (not shown). Partitioning of positive and negative droplets was also impacted in enrichments broths generated from leafy-green produce such as spinach or kale (**Figure 7C**), where dark green pigments were observed in the enrichment broths.

Despite apparent inefficiencies with the use of intact cells in the MuSIC ddPCR assay, the application of the percent linkage value, with positives defined as samples with greater than 10% linkage between *stx* and *eae* targets, provided a robust metric for distinguishing EHEC-positive samples from mixed cultures containing targets in different cells, even in samples containing high levels of non-target bacteria (e.g., 10,000-fold excess). In samples of mixed strains that would have generated false positives by traditional PCR-based methods, linkage between the two targets was extremely low, less than 0.5% in most samples. The use of undiluted food enrichments had some impact on the performance of the assay. For example, the higher degree of variability in percent linkage values for EHEC mixed at 1:1000 with food enrichment broths relative to the 1:100 mixtures (**Figure 7A**) may be due to the lower dilution used to achieve the optimal concentration of EHEC in the 1:1000 samples. Nonetheless, even in these samples, accurate identification of EHEC was achieved. Use of a positive control will be important for evaluating the impact of inhibition in different food matrices. This proof-of-concept study shows promising results for the use of MuSIC ddPCR with food enrichments broths containing food debris and high relative proportions of background bacteria. The performance of the method was robust in food-enrichment samples where concentration of target cells was between 50 and 2500 cfu/reaction. While this is a limited dynamic range for detection compared to other PCR-based approaches, in practice testing of up to three dilutions of the enrichment broths will enable accurate detection of EHEC-positive samples.

False-positive detection of EHEC is a significant challenge for food-microbiology laboratories (Krause et al., 2005; Shelton et al., 2006; Feng et al., 2010; Alonso et al., 2011; Delannoy et al., 2016). For example, in a study including 1739 beef enrichment broths, approximately half of the 180 enrichment broths positive for *stx* and *eae* markers were false-positives, and did not contain EHEC (Delannoy et al., 2016). The work required for the analysis of these false-positive samples is onerous, as many colonies would need to be screened to confirm either the presence or absence of EHEC in the sample. To achieve higher specificity, the Canadian (Blais et al., 2014b), ISO/CEN TS13136:2012 Technical specification (ISO, 2012) and the US MLG5B.05 (USDA-FSIS, 2014) methods also detect *O*-serogroup specific markers for priority serovars, that vary among jurisdictions. Unfortunately, serogroup markers can also be present in non-pathogenic strains, and not all of the clinically important EHEC fall within the priority serogroups (EFSA, 2013). New screening methods that use additional genes, associated with typical EHEC, have been effective in reducing false-positive detection of EHEC in food enrichment broths (Delannoy et al., 2013, 2016), but these rely on the detection of a number of different genes known to be associated with EHEC, none of which (individually) are present in all EHEC variants. Specific detection of samples containing typical EHEC, which are *eae*- and *stx*-positive, would enable identification of emerging pathotypes in foods.

The MuSIC ddPCR EHEC assay enables accurate detection of EHEC in enrichment broths based on detection of two diagnostic genetic markers (*stx* and *eae*) and determination that these occur in a single cell. While this method is somewhat more complex than standard PCR approaches, the benefit in reducing false-positive detection of EHEC outweigh the challenges associated with the implementation of the method. A more extensive validation of the assay with priority food types (e.g., leafy-green produce and raw meat) will be undertaken to enable deployment to food-testing laboratories. Integration of additional targets such as the *stx2f* toxin subtype and/or the *aaiC* and *aggR* markers associated with the seropathotype responsible for the 2011 European O104 outbreak (EFSA, 2013) may also be of value. The reduction in false-positive detection of EHEC associated with the application of the MuSIC ddPCR EHEC assay will enable high throughput screening for EHEC in food samples, ultimately reducing consumer exposure to EHEC-contaminated products.

## Author Contributions

Conceived and designed the experiments: TM, CC, BB, and AW. Performed the experiments: TM. Analyzed the data: TM and CC. Contributed reagents, materials, analysis tools: CC and BB. Wrote the paper: TM, CC, BB, and AW.

## Conflict of Interest Statement

The authors declare that the research was conducted in the absence of any commercial or financial relationships that could be construed as a potential conflict of interest.

## Footnotes

1 https://cge.cbs.dtu.dk/services/data.php
