# Supplementary figures and images for: Multiplexed Single Intact Cell Droplet Digital PCR (MuSIC ddPCR) Method for Specific Detection of Enterohemorrhagic E. coli (EHEC) in Food Enrichment Cultures
Source: Front Microbiol. 2017 Mar 2;8:332. doi: 10.3389/fmicb.2017.00332 (PMC5332415; doi:10.3389/fmicb.2017.00332)

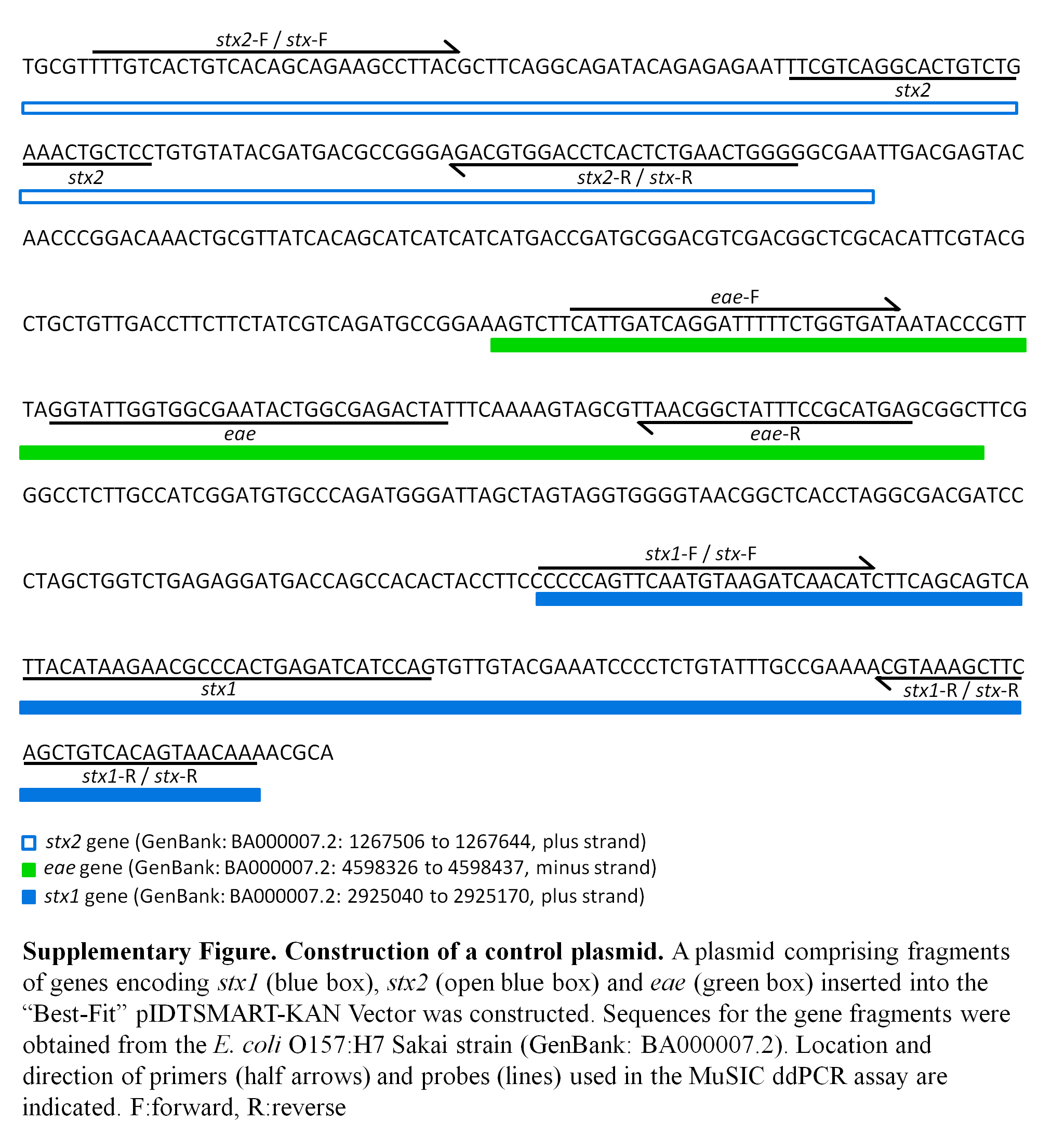

Supplement: Supplementary file 1 [file Image_1.TIF]
